# Role of long non-coding RNAs in rice reproductive development

**DOI:** 10.3389/fpls.2022.1040366

**Published:** 2022-11-15

**Authors:** Saeid Babaei, Mohan B. Singh, Prem L. Bhalla

**Affiliations:** Plant Molecular Biology and Biotechnology Laboratory, Faculty of Veterinary and Agricultural Sciences, The University of Melbourne, Melbourne, VIC, Australia

**Keywords:** lncRNAs, circRNAs, rice, reproduction, anther, pollen, seed development

## Abstract

Rice is a staple crop, feeding over half of the global population. The future demand of population growth and climate change requires substantial rice improvement. Recent advances in rice genomics have highlighted the vital role of the non-coding part of the genome. The protein-coding regions account for only a tiny portion of the eukaryotic genome, and most of the genomic regions transcribe copious amounts of non-coding RNAs. Of these, the long non-coding RNAs, including linear non-coding RNAs (lncRNAs) and circular non-coding RNAs (circRNAs), have been shown to play critical roles in various developmental processes by regulating the expression of genes and functions of proteins at transcriptional, post-transcriptional and post-translational levels. With the advances in next-generation sequencing technologies, a substantial number of long non-coding RNAs have been found to be expressed in plant reproductive organs in a cell- and tissue-specific manner suggesting their reproductive development-related functions. Accumulating evidence points towards the critical role of these non-coding RNAs in flowering, anther, and pollen development, ovule and seed development and photoperiod and temperature regulation of male fertility. In this mini review, we provide a brief overview of the role of the linear and circular long non-coding RNAs in rice reproductive development and control of fertility and crop yield.

## Introduction

Continuous advances in high throughput sequencing technologies coupled with the development of efficient bioinformatics tools have revealed the complexity of dynamic gene expression in plant cells (Kaufmann and Chen, 2017; García-Gómez et al., 2020; Alvarez et al., 2021). Transcriptional and post-transcriptional processes can generate diverse RNA molecules with or without protein-coding potential. Protein coding RNAs, or messenger RNAs (mRNAs), that carry the genetic code that can be translated to proteins comprise less than 2% of the transcriptome in a cell. The vast majority of RNAs are non-protein coding or non-coding RNAs (ncRNAs) with the potential to regulate essential biological functions in various processes. Non-coding RNAs are diverse but based on their size and form; they can be classified into small ncRNAs, long non-coding RNAs (lncRNA), and circular RNAs (circRNAs) (Jarroux et al., 2017; Kaikkonen and Adelman, 2018; Panni et al., 2020). Recent studies have indicated that a subset of ncRNAs can encode small peptides or polypeptides with known or unknown functions (Choi et al., 2019; Kong et al., 2020).

LncRNAs are defined as transcripts with a length of over 200 nucleotides with no apparent protein-coding potential (Waseem et al., 2020). Based on the functional studies during the past decade, lncRNAs can be categorized as RNA molecules that exert their roles with their nucleotide sequences and structure independent of their potential genetic code (Wierzbicki et al., 2021). LncRNAs can be classified based on their genomic locations with coding regions: including intronic, intergenic, sense, and antisense directions lncRNAs. Compared to protein-coding RNAs, lncRNAs have certain unique characteristics, such as less sequence conservation, shorter length, and more tissue-specific expression (Golicz et al., 2018; Rai et al., 2019). LncRNAs modulate the expression of their neighboring or distal genes through various mechanisms (Lucero et al., 2021). For example, LncRNA ELF18-INDUCED LONG NONCODING RNA1 (ELENA1), which is expressed from the promoter region of the PATHOGENESIS-RELATED 1 (PR1) gene in Arabidopsis has been shown to activate and enhance the expression of PR1 in response to pathogen elicitors by directly interacting with core transcriptional machinery at the PR1 promoter (Seo et al., 2017). LncRNA ALTERNATIVE SPLICING COMPETITOR (ASCO) was shown to interact with nuclear speckle RNA-binding proteins (NSRs: alternative splicing regulators) to regulate gene expression by modulating the alternative splicing patterns of NSR target genes during plant development (Bardou et al., 2014). LncRNAs can also regulate the expression of distal genes by forming DNA-RNA duplexes or R-loops. APOLO (AUXIN-REGULATED PROMOTER LOOP) is an example of a lncRNA that can regulate the expression of several distal and independent genes in the Arabidopsis genome by targeting short complementary sequences and forming R-loops (Ariel et al., 2020). APOLO is also associated with regulating gene expression in response to Auxin through the formation of chromosome looping and epigenetic modifications (Ariel et al., 2014). LncRNAs can also control the gene expression either by acting as miRNA target mimicry or as precursors of miRNAs and other small RNAs such as phased secondary small interfering RNAs (phasiRNAs). In the target mimicry mechanism, lncRNAs perform their role by sponging miRNAs and sequestering them from repressing their mRNA targets (Borah et al., 2018). As the small RNA substrate, lncRNAs are cleaved by Argonaute complexes to generate miRNAs or phasiRNAs (Meng et al., 2021).

CircRNAs are another class of regulatory lncRNA molecules that arise from an uncanonical type of splicing named back-splicing, during which the spliceosome covalently joins an upstream 3′ splice site to a downstream 5′ splice site to form circle RNAs with no 5’ caps or 3’ poly-A tails (Xiao et al., 2020). Like linear lncRNA, circRNAs have tissue-specific expression and can be transcribed from various genomic regions, including exons, introns, and intergenic regions (Guria et al., 2020). However, many identified circRNAs in most plant species were composed of exonic regions (Chu et al., 2020). CircRNAs mostly have a low abundance in the cell, but in some cases, they can accumulate to a high level, mostly due to their resistance to exonucleases (Liu and Chen, 2022). Studies in humans and animals have reported the involvement of circRNAs in many molecular processes, such as transcription, alternative splicing, translation, and miRNA sponging (Xiao et al., 2020; Guria et al., 2020; Liu and Chen, 2022). For example, exon-intron circRNAs abundant in the nucleus of human cells were associated with RNA polymerase II machinery and reported to positively regulate the expression of their parent genes through *cis*-acting regulatory roles (Li et al., 2015). CircRNAs can also regulate translation and cell division by interacting with proteins. CircRNA Poly(A)-Binding Protein Nuclear 1 (circPABPN1) has been shown to regulate the translation of its cognate mRNA by interacting with RNA-binding protein Human antigen R (HuR). CircPABPN1 could bind HuR and prevent HuR from binding PABPN1 mRNA, which resulted in the reduced translation of PABPN1 mRNA (Abdelmohsen et al., 2017). MiRNA sponging is probably one of the well-known functions of circRNAs in humans and animals. For example, CDR1as contains over 70 binding sites for miR-7, and the overexpression of CDR1as in human cells suppressed the activity of miR-7 significantly, indicating this circRNAs as miRNA sponging (Hansen et al., 2013; Memczak et al., 2013). In plants, circRNAs have been reported to be expressed at almost all stages of growth and development (Zhao et al., 2019). Although the exact molecular function of most identified circRNAs in plants remains to be explored, previous studies have suggested that circRNAs could have important functions during plant development and in response to exogenous stimuli (Zhao et al., 2019; Zhang et al., 2020). For example, transcriptomic studies revealed differential expression of circRNAs in various tissues and developmental stages such as root, stem, leaf, and flowers, as well as in response to pathogens and biotic stresses such as drought, salinity, cold, and nutrient deficiency (Zhang et al., 2020). Plant circRNAs have also been shown to form R-loop to regulate gene expression in Arabidopsis (Conn et al., 2017) and regulate chromatin organization in maize (Liu et al., 2020). Although various studies have proposed that plant circRNAs can act as miRNA sponges, very few experimental evidence is available (Chu et al., 2020).

**Figure 1:**
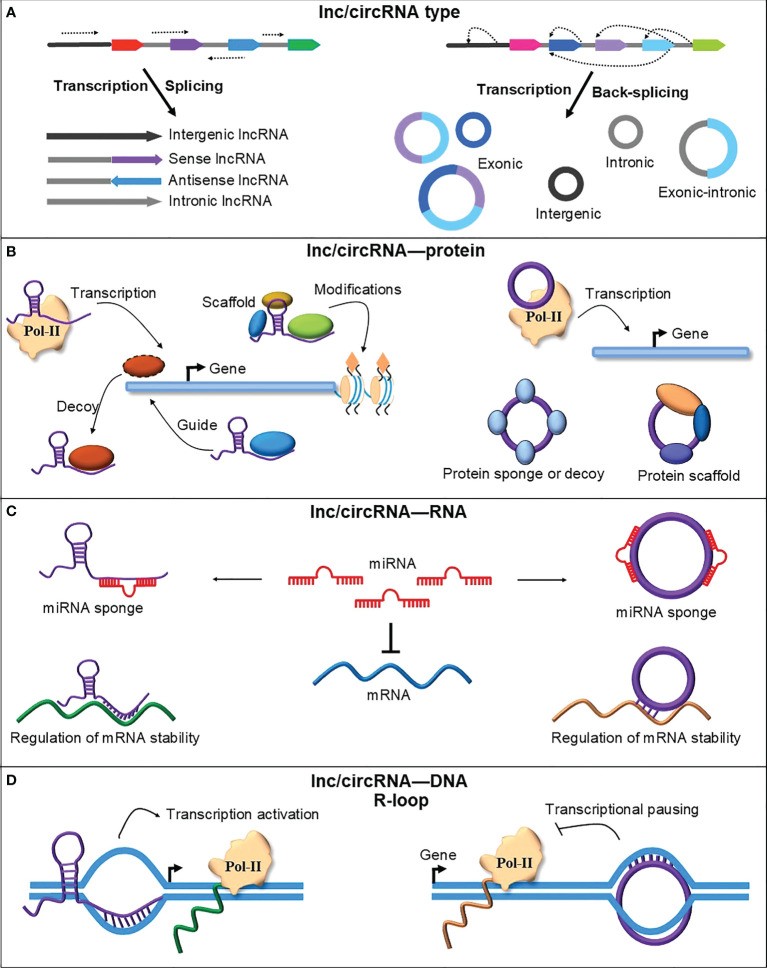
A schematic representation of lncRNAs and circRNAs types and their mode of action. **(A)** RNA polymerase II transcription and canonical splicing produce different types of lncRNAs from genic and intergenic regions. CircRNAs are also transcribed using RNA polymerase II but spliced *via* an uncanonical type of splicing named backsplicing. CircRNAs are produced from genic and intergenic regions but mainly from the circularization of one or more exons. **(B)** LncRNAs and circRNAs could interact with different proteins to perform their function. They can interact with RNA Polymerase II (Pol-II) components to regulate transcription. LncRNAs can recruit different proteins, such as transcription factors and chromatin modifiers, to guide them to their place of activity or titrate them away from their site of action to signal the activation or deactivation of gene expression. CircRNA can also sponge or scaffold various proteins to perform their cellular function, such as regulating gene expression, translation, and cell division (Huang et al., 2020). **(C)** LncRNAs and circRNAs can bind to other RNAs and regulate gene expression. LncRNAs and circRNAs block or reduce the negative effect of miRNAs on target mRNAs by sponging miRNAs. LncRNAs and circRNAs can also modulate the stability and translation of mRNAs by directly binding their target mRNAs on the recognition site of miRNAs or RNA-binding proteins (Sebastian-delaCruz et al., 2021; Wu et al., 2022). **(D)** LncRNAs and circRNAs can directly bind DNA and form an RNA-DNA hybrid or R-loop. By forming the R-loops, lncRNAs and circRNAs regulate the transcription of their parent or adjacent genes by affecting the RNA polymerase II transcriptional machinery.

Plant reproduction is one of the most important stages as its success determines crop yield. The switch from the vegetative to reproductive phase of a plant occurs in response to internal (age) and external signals (photoperiod or temperature), leading to changes in the overall gene expression during reproductive development (Wong et al., 2013; Luo et al., 2021). Studies have identified lncRNAs and circRNAs as the key regulatory elements during plant reproduction (Yamaguchi e Abe, 2012; Golicz et al., 2018; Zhao et al., 2019) as these are reported to perform critical regulatory roles such as chromatin remodeling, transcription, molecular scaffolding and sequestering proteins and RNAs (Ransohoff et al., 2018; Yu et al., 2019; Budak et al., 2020) (**Figure 1**).

In this review, we summarize the recent findings on the role of lncRNAs and circRNAs in rice reproductive development. Rice, one of the major staple crops consumed by over half the world’s population. Rice is grown in subtropical and tropical areas. Rice is the source of calories and nutrition in many developing countries. Rice production must be increased by 1.5x by 2030 to meet the growing population’s demand. Rice crop yield is sensitive to the environment and climate change. Rice improvement through genomic and modern technology is much needed to meet the future demand.

## LncRNAs regulate flowering

Flowering starts with transforming shoot apical meristem into inflorescence meristem. The floral organs then originate from the floral meristem that form lateral organs such as carpel, anther, and pollen (Itoh et al., 2005). In Arabidopsis, three lncRNAs termed COLDAIR (cold assisted intronic noncoding RNA), COOLAIR (cold-induced long antisense intragenic RNA), and COLDWRAP (cold of winter-induced noncoding RNA from the promoter) have been shown to play a role in vernalization and floral transition by epigenetically modify the chromatin and silence the repressor FLC (Heo and Sung, 2011; Csorba et al., 2014; Kim and Sung, 2017).

**Figure 2:**
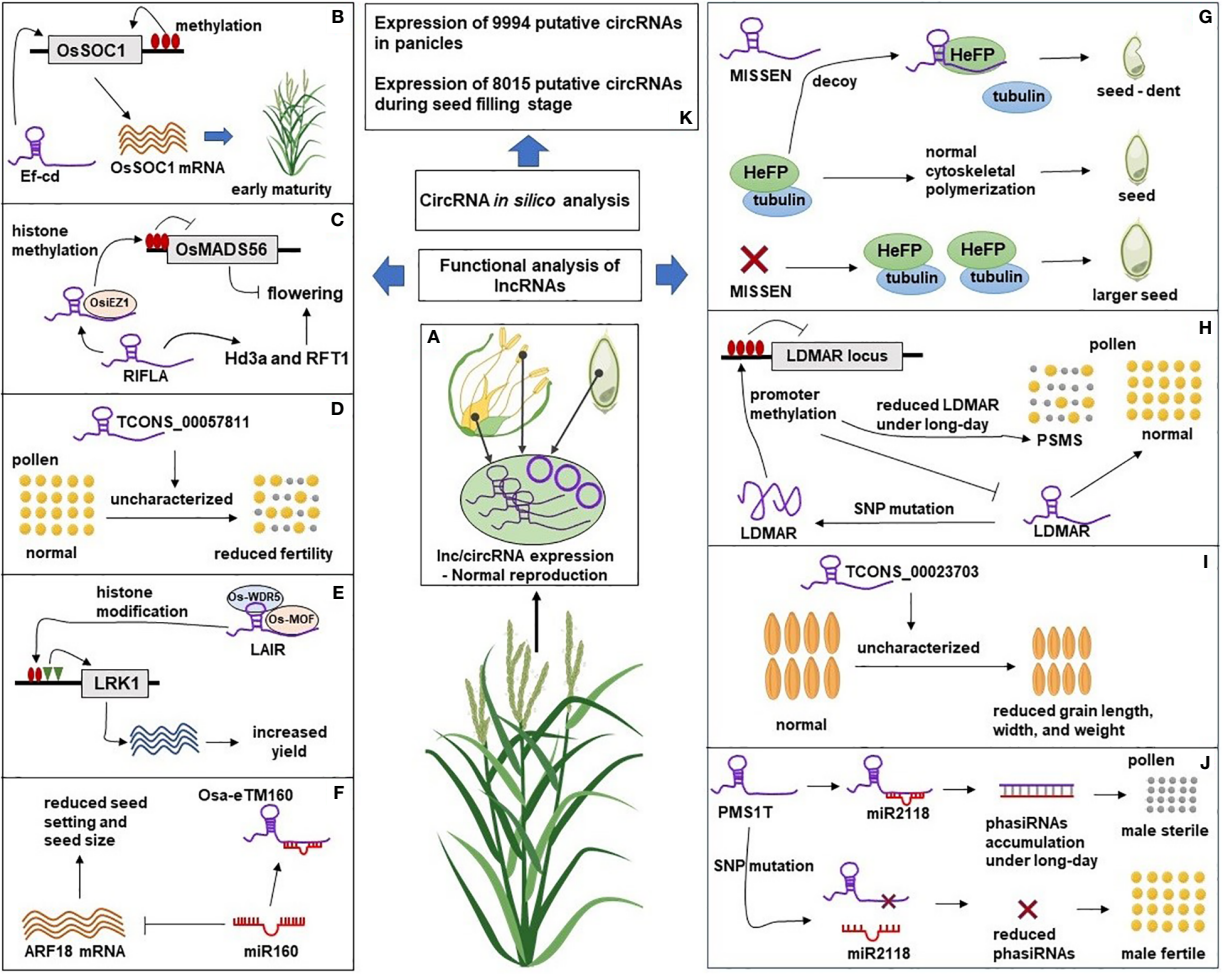
A Schematic representation of lncRNAs and circRNAs roles in rice reproduction. **(A)** The natural expression of lncRNAs and circRNAs in reproductive tissues leads to normal rice reproductive development. **(B)** LncRNA Ef-cd could promote the expression of flowering gene OsSOC1 leading to early flowering. **(C)** Overexpression of lncRNA RIFLA induced the expression of flowering genes Hd3a and RFT1 and inhibited the expression of floral repressor gene OsMADS56 by recruiting histone methyltransferase OsiEZ1 at OsMADS56 locus. **(D)** Overexpression of lncRNA TCONS_00057811 caused reduced pollen fertility and seed set in the transgenic plant through an uncharacterized mechanism. **(E)** Overexpression of lncRNA LAIR promoted H3K4me3 and H4K16ac at LRK1 locus by recruiting histone modifying proteins (OsMOF and OsWDR5) resulting in increased expression of LRK, a gene family related to rice yield. **(F)** Overexpression of lncRNA Osa-eTM sponged miR160 and reduced its negative effect on ARF18 mRNA leading to reduced seed setting and seed size in transgenic lines. **(G)** HeFP, a helicase family protein, interacts with tubulin to regulate cytoskeletal polymerization during endosperm development. LncRNA MISSEN regulates HeFP ensuring normal endosperm development. Overexpression of MISSEN could interrupt the HeFP-tubulin interaction resulting in dents and bulges in developing seeds. Down-regulation of MISSEN removed the activity of MISSEN on HeFP, resulting in the production of larger seeds. **(H)** Expression of lncRNA LDMAR is required for pollen development. Change in the secondary structure of LDMAR caused by SNP mutation increased methylation at the LDMAR locus and reduced LDMAR expression specificity under long-day photoperiod and PSMS. **(I)** Down-regulation of lncRNA TCONS_00023703 resulted in reduced grain length, width, and 1000-grain weight through an unknown mechanism. **(J)** LncRNA PMS1T is the precursor of 21nt-phasiRNAs, which are involved in photoperiod-sensitive male sterility (PSMS) under the long-day condition. Down-regulation of phasiRNAs through SNP mutation near their binding site on PMS1T resulted in male fertility. **(K)** *In silico* analysis identified the expression of circRNAs in rice reproductive tissues. Functional experiments are needed to reveal the mechanistic roles of circRNAs during rice reproductive development. The Rice plant was “Created with BioRender.com”.

LncRNAs have been shown to regulate flowering and different stages of reproductive development in rice (**Table 1**, **Figure 2**). Recently Shin et al. (2022) have reported that OsMADS56-mediated flowering control is strictly regulated by an lncRNA, RIFLA (Rice Flowering Associated), originating from the first intron of the *OsMADS56* gene. OsMADS56 is a MIKC-type MADS-box protein that acts as a floral repressor. Overexpression of OsMADS56 in transgenic rice delays the onset of flowering (Ryu et al., 2009). Overexpression of lncRNA, RIFLA inhibited the expression of *OsMADS56* gene but enhanced the expression of flowering inducers genes such as Hd3a (Heading date 3a) and RFT1 (RICE FLOWERING LOCUS T 1), resulting in the early flowering phenotype (Shin et al., 2022).

**Table 1:** A list of lncRNAs and circRNAs studied during rice reproduction.

lncRNA name	Transcribed region	Expression specificity	lncRNA function	Mechanism of action	reference
**nTCONS_00057811**	–	Anther specific expression	Overexpression resulted in reduced pollen fertility and seed set	Uncharacterized	(Li et al., 2020)
**RIFLA**	The first intron of OsMADS56	Suppresses the expression of the parent gene	Overexpression resulted in early flowering by inhibiting the OsMADS56 gene (floral repressor) and inducing Hd3a and RFT1 genes (floral inducers)	Histone methylation (H3K27me3) by recruiting histone methyltransferase OsiEZ1	(Shin et al., 2022)
**LDMAR**	Sense strand relative to transcript AK111270	Anther specific expression at vacuolated pollen cell stage required for normal pollen growth and development under long-day photoperiod	Down-regulation of LDMAR activated programmed cell death in developing anthers under long-day photoperiod and caused photoperiod-sensitive male sterility	Altered secondary structure of LDMAR caused by SNP mutation, increased methylation in the promoter region of LDMAR	(Ding et al., 2012)
**LAIR**	The antisense strand of neighboring region of the LRK cluster gene	High level of expression in three-leaf stage shoot and reproductive tissues	Overexpression positively regulated the expression of many LRK genes and increased seed production	Activation of the LRK1 gene by promoting H3K4me3 and H4K16ac. Binds untranslated regions and histone modifying proteins (OsMOF and OsWDR5) to regulate the epigenetic changes	(Wang et al., 2018)
**Osa-eTM160**	Intergenic	High and specific expression in the early reproductive stage	Osa-eTM attenuated the repression of osa-miR160 on osa-ARF18 mRNAs through target mimicry manner to affect seed setting and seed size	Targets miR160 to regulate the expression of Osa-ARF18 mRNA during the anther development	(Wang et al., 2017)
**MISSEN**	Intergenic	Specific expression in reproductive tissues	Overexpression of MISSEN resulted in dents and bulges in the seed. The down-expression of MISSEN resulted in larger seeds by increasing nuclear division and endosperm cellularization	Inhibits the interaction of HeFP (a helicase family protein) with tubulin and causes abnormal cytoskeletal polymerization during endosperm development	(Zhou et al., 2021b)
**TCONS_00023703**	–	High expression during seed development	RNAi-based knock-down resulted in reduced grain length, width, and 1000-grain weight	Uncharacterized	(Zhao et al., 2020)
**Ef-cd**	*EF-cd*, lncRNA transcribed from the antisense strand of flowering activator *OsSOC1* gene	High expression in panicles	*Ef-cd* lncRNA enhanced the expression of *OsSOC1*, leading to early maturity without affecting the rice yield.Ef-cd improved nitrogen utilization and promoted photosynthesis rate	Ef-cd can potentially promote the level of methylation (H3K36me3) in the OsSOC1 locus	(Fang et al., 2019)
**PMS1T**	*Pms1* locus encodes lncRNA s	PMS1T has preferential expression in young panicles	The phasiRNAs regulate photoperiod-sensitive male sterility (PSMS) in riceSNP in PMS1T near the miR2118 recognition site changed the accumulation of phasiRNAs and fertility	Functions as a precursor of 21-nt phasiRNAs when targeted by miR2118	(Fan et al., 2016)
***Os06circ02797***	*Intron of Os06g04610*	–	Sequesters miR408	Uncharacterized	(Zhou et al., 2021a)
***Total of 8015 putative circRNAs***	*-*	Rice grains at filling stage	Some circRNAs could potentially act as miRNA sponges	Uncharacterized	(Chen et al., 2022)
***Total of 9994 putative circRNAs***	*-*	Panicles	Some circRNAs could potentially act as miRNA sponges.Gene ontology analysis suggested role of parent gene of circRNAs in various biological processes such as metabolic, developmental, and reproductive processes	Uncharacterized	(Wang et al., 2019)

## LncRNAs regulate pollen development and male-fertility

Pollen development within the anther locules requires the expression and regulation of hundreds of genes during which mature fertile pollen is produced (Singh & Bhalla, 2007; Singh et al., 2008) Pollen, a haploid male gametophyte, contains male germ cells formed from meiocytes (or pollen mother cells) after meiosis, followed by asymmetric mitotic division to give rise to a larger vegetative cell and a smaller generative cell (Haerizadeh et al., 2006; Golicz et al., 2021). Two sperm cells are produced from the generative cell. The transcriptional repertoire of vegetative, generative and sperm cells is quite distinct (2007; Okada et al., 2005; Sharma et al., 2011; Russell et al., 2012). The knowledge of gene regulatory control during these development stages is limited. The involvement of lncRNAs in pollen development has been revealed in some plant species such as *Brassica rapa* (Huang et al., 2018), cotton (Li et al., 2019) and tomato (Lamin-Samu et al., 2022). In rice, systematic identification studies have demonstrated a highly enriched and specific expression of lncRNAs in anthers, pointing to the involvement of lncRNA in pollen development. Anther-specific lncRNAs have been suggested to be involved in germ cell development or meiosis, potentially by acting as competing endogenous RNAs (ceRNAs) to sequester flowering-related miRNAs such as miR160 or miR164 from their target genes (Zhang et al., 2014). Studies in autotetraploid rice have revealed differential expression of 444 lncRNAs during meiosis in the anther and ovary. Some lncRNAs, such as lncRNAs derived from TCONS_00057811, TCONS_00055980 and TCONS_00130461, showed high levels of tissue-specific expression in meiotic stage anthers. Overexpression of TCONS_00057811 (lncRNA57811) reduced pollen fertility and seed setting in transgenic lines compared with wild type (Li et al., 2020).

LncRNAs have been demonstrated to regulate male fertility and pollen development in rice (**Table 1**). For example, in rice growing under long-day photoperiod conditions, the expression of LDMAR (long-day specific male-fertility–associated RNA), a lncRNA with a length of about 1200 nt, is essential for normal pollen development. However, a single nucleotide mutation altered the secondary structure of LDMAR, which subsequently promoted the methylation of the LDMAR putative promoter and decreased its expression. This reduction in LDMAR expression, which was more observed explicitly during the long-day photoperiod, activated programmed cell death in developing anthers and caused photoperiod-sensitive male sterility (Ding et al., 2012).

LncRNAs could also be involved in fertility transition in rice. Wuxiang S is a rice line that produces a male-sterile phenotype when the temperature is > 24°C and the day length is > 12 hours. However, Wuxiang S can become male-fertile and have normal anthers when plants (with panicle length ~1 cm) are transferred to a growth condition under 12 hours day photoperiod with a temperature of about 21°C. A comparison of RNA sequencing and bioinformatic analysis between the Wuxiang S male-sterile line and its photo-thermo transitioned male-fertile counterpart revealed differential expression of 622 lncRNAs during different stages of pollen development. Functional analysis of cis and trans targets of lncRNAs revealed genes involved in pollen development-related processes, including regulation of carbohydrate metabolism, starch accumulation, lipid metabolism, formation of anther cuticle and pollen exine, regulation of rice tapetum degeneration, and hormone signal transduction (Wang et al., 2021).

LncRNAs can also be expressed as a precursor of miRNAs and function during rice pollen development. It has been shown that over 700 long intergenic non-coding RNAs (lincRNAs) were expressed specifically in inflorescences. These reproductive stage-specific lincRNAs contain a complementary sequence for miR2118 which could cleave through DICER-LIKE4 (DCL4) protein at the miR2118 site to generate 21-nucleotide phased small interfering RNAs (phasiRNAs) in the germ cells. PhasiRNAs are associated with MEIOSIS ARRESTED AT LEPTOTENE1 (MEL1), which is a rice Argonaute (AGO) protein that regulates the progression of meiosis and development of germ cells in male and female organs (Komiya et al., 2014). Rice *mel1* mutants show irregular development of germ cells, such as prevention of the chromosome condensation at the early meiosis and anomalous vacuolation of pollen mother cells (Nonomura et al., 2007). It is proposed that the MEL1-phasiRNAs interactions could modulate target genes involved in maintaining the germ-cell identity and its normal development in the pre-miotic to miotic stages (Komiya et al., 2014). *PMS1T* transcribed from Pms1(photoperiod-sensitive genic male sterility 1) locus is a particular example of lncRNAs that function as a precursor of phasiRNAs when targeted by miR2118. It has been observed that under the long-day condition, *PMS1T* accumulate in the photoperiod-sensitive male sterility lines, and a single SNP in *PMS1T* nearby the miR2118 recognition site can alter fertility through changing phasiRNAs accumulation (Fan et al., 2016).

## LncRNAs regulate ovule development and seed production

Ovules are female reproductive organs with distinct gene expressions that, after fertilization, develop into seeds (Shi and Yang, 2011). In rice, differential expression of lncRNAs has been observed between female-sterile and female-fertile rice during different stages of ovule development, suggesting the role of lncRNAs in ovule development and abortion of female gametophyte (Liu et al., 2019). LncRNA XLOC_057324, with high expression in young panicles and pistils, has also been suggested to play a role in flower development and sexual reproduction. Mutant rice lines with a T-DNA insertion in XLOC_057324 showed an early flowering phenotype with a reduced fertility rate (Zhang et al., 2006; Zhang et al., 2014). Early flowering rice cultivars generally have lower seed yields than late flowering cultivars because of the shorter maturity duration. Studies have demonstrated that the innate variation in Ef-cd (early flowering-completely dominant) gene can decrease the maturity duration without affecting the rice yield. Ef-cd is an antisense lncRNA overlapping the *Os-SOC1* gene, and this lncRNA can enhance the expression of the Os-SOC1 gene. Further analysis revealed that Ef-cd improves the yield in early flowering rice by promoting nitrogen utilization and photosynthesis (Fang et al., 2019).

The long non-coding RNAs also regulate seed development in rice. LncRNA MISSEN (mis-shapen endosperm), known previously as XLOC_057324, has been demonstrated to inhibit HeFP (a helicase family protein) from interaction with tubulin during endosperm development causing abnormal cytoskeletal polymerization. Overexpression of MISSEN suppressed normal development of endosperm, resulting in dents and bulges in the seed, while lines with reduced expression of MISSEN showed increased nuclear division and endosperm cellularization resulting in larger seeds compared with wild type. It is worth noting that histone methylation inhibits MISSEN expression after pollination. (Zhou et al., 2021b). LncRNAs also regulate seed development through lncRNA-miRNA-mRNA interactions. LncRNA Osa-eTM160 (eTM: endogenous target mimic) has been shown to regulate the expression of Osa-ARF18 mRNAs by targeting Osa-miR160. Osa-ARF18 mRNAs and Osa-miR160 have high expression in early anther developmental stages. Overexpression of Osa-eTM160 reduced the activity of Osa-miR160 and caused a reduced seed setting rate and seed size in transgenic lines (Wang et al., 2017). Driving epigenetic changes is another way that lncRNAs regulate seed development in rice. The involvement of lncRNA LAIR (leucine-rich repeat receptor kinase antisense intergenic RNA) transcribed from neighboring regions of the LRK (leucine-rich repeat receptor kinase) cluster gene has been demonstrated during seed development. Overexpression of LAIR could positively regulate the expression of many LRK genes leading to increased seed production. Further, it was revealed that LAIR could bind 5′ and 3′ untranslated regions of the LRK1 gene and histone modification proteins such as Os-MOF and Os-WDR5. LAIR then activates the LRK1 gene by promoting H3K4me3 and H4K16ac modifications (Wang et al., 2018).

Differential expression of lncRNAs and alternative splicing have also been observed during seed development in rice (Kiegle et al., 2018; Zhao et al., 2020). For instance, RNA sequencing studies on developing seeds (three and seven days after pollination) revealed 482 lncRNAs with differential expression patterns. Transgenic rice lines with RNAi-based downregulation of expression of lncRNA TCONS_00023703 showed a significant reduction in grain length, width, and 1000-grain weight (Zhao et al., 2020). In addition, more exon inclusion in lncRNAs has been observed in embryo; a such example is LOC9270896 which potentially produces 20 variant transcripts with a length between 3,714 to 4,616 bp, but the predominate variant in the embryo was a short variant (593 bp) that all three introns were spliced out (Kiegle et al., 2018).

## CircRNAs are involved in reproductive development

Genome-wide RNA expression analysis has revealed that circRNAs show preferential expression in different plant tissues. For example, of 5372 identified circRNAs in soybean, 2647, 1644, and 484 circRNAs were expressed preferentially in the root, stem, and leaf tissues, respectively, and the remaining circRNAs were common between two or three tissues (Zhao et al., 2017). Similar results have been found when circRNA expression was compared between root, leaf, and flower tissues in Arabidopsis (Philips et al., 2020). A comprehensive survey of rice transcriptome identified 15,122 lncRNAs and 7902 circRNAs in root, leaf and panicle tissues (Zhou et al., 2021a). Both lncRNAs and circRNAs are expressed in a highly tissue-specific manner, with panicle-specific expression being the most pronounced. Moreover, the tissue-specific expression of circRNAs was much higher compared to lncRNAs. GO enrichment for parental genes of circRNAs revealed the involvement of circRNAs in reproductive and post-embryonic development in a panicle.

Further, circRNA Os06circ02797 generated by locus Os06g04610 harbors binding sites for OsmiRNA-408. OsmiRNA408 positively enhances photosynthesis and regulates grain yield in rice increasing panicle branches and grain number (Zhang et al., 2017). Zhou et al. (2021a) produced CRISPR‐Cas9 edited stable transgenic rice lines with deleted Os*06circ02797* locus. The seedlings of *Os06circ02797* null mutant demonstrated rapid growth phenotype following seed germination. Further analysis revealed the functioning of circRNA-miRNA-mRNA regulatory network where *Os06circ02797* binds and sequesters OsMIR408.

The circular RNAs are also involved in pollen development. For example, in *Brassica rapa*, differential expression of circRNAs during pollen development has been observed. Functional analysis suggested their potential roles in pollen-related developmental processes such as cell cycle, meiosis/mitotic cell division, and polysaccharide biosynthesis (Babaei et al., 2021). In rice, circRNAs were found to be involved in pollen development and fertility transition in photo-thermosensitive genic male sterile lines. Transcriptome comparison of young panicles from male sterile and fertile lines at different developmental stages detected specific expression patterns of circRNAs during pollen development and fertility transition. Functional annotation analysis suggested that circRNAs could have important roles in the regulation of cell differentiation, cell division, regulation of hormone levels, response to temperature stimulus, and floral organ development. CircRNAs could also act as ceRNAs by targeting miR399, an ambient temperature-responsive flowering regulator (Teotia and Tang, 2015; Wang et al., 2019).

CircRNAs are also reported to be involved in grain filling in rice. For instance, in a recent study, over 8000 putative circRNAs were identified during different stages of seed development. *In silico* analysis suggested that while some circRNAs could act as miRNA decoys targeting miR-164 and miR-398, other circRNAs could function in various processes such as carbohydrate metabolic processes, embryo development, and lipid transport (Chen et al., 2022).

## Concluding remarks

Reproduction, a critical developmental process, determines yield and productivity of crop plants. Transcriptome studies using next-generation sequencing experiments have revealed the differential expression pattern of lncRNAs and circRNAs and their wide distribution during reproductive processes in rice. Although the importance and diverse potential functions of lncRNAs and circRNAs have been proposed using *in silico* analysis, the molecular function of only a few reproductive-related lncRNAs has been elucidated to date. The involvement of lncRNAs in flowering, pollen development, fertility, seed development, and seed yield add another layer of complexity to plant gene regulation during growth and development. However, the journey toward understanding the role of lncRNAs and circRNAs in plant reproduction has just begun. We expect more functional studies unraveling reproductive processes to be published in the coming years.

## Author contributions

All authors listed have made a substantial, direct, and intellectual contribution to the work, and approved it for publication.

## Funding

SB held the University of Melbourne Postgraduate Scholarship during this study.

## Conflict of interest

The authors declare that the research was conducted in the absence of any commercial or financial relationships that could be construed as a potential conflict of interest.

## Publisher’s note

All claims expressed in this article are solely those of the authors and do not necessarily represent those of their affiliated organizations, or those of the publisher, the editors and the reviewers. Any product that may be evaluated in this article, or claim that may be made by its manufacturer, is not guaranteed or endorsed by the publisher.

## References

[B1] Abdelmohsen K. Panda A. C. Munk R. Grammatikakis I. Dudekula D. B. De S.. (2017). Identification of HuR target circular RNAs uncovers suppression of PABPN1 translation by CircPABPN1. RNA Biol. 14 (3), 361–369. doi: 10.1080/15476286.2017.1279788 28080204 PMC5367248

[B2] Alvarez J. M. Brooks M. D. Swift J. Coruzzi G. M. (2021). Time-based systems biology approaches to capture and model dynamic gene regulatory networks. Annu. Rev. Plant Biol. 72 (1), 105–131. doi: 10.1146/annurev-arplant-081320-090914 33667112 PMC9312366

[B3] Ariel F. Jegu T. Latrasse D. Romero-Barrios N. Christ A. Benhamed M.. (2014). Noncoding transcription by alternative RNA polymerases dynamically regulates an auxin-driven chromatin loop. Mol. Cell 55 (3), 383–396. doi: 10.1016/j.molcel.2014.06.011 25018019

[B4] Ariel F. Lucero L. Christ A. Mammarella M. F. Jegu T. Veluchamy A.. (2020). R-loop mediated trans action of the APOLO long noncoding RNA. Mol. Cell 77 (5), 1055–1065. doi: 10.1016/j.molcel.2019.12.015 31952990

[B5] Babaei S. Singh M. B. Bhalla P. L. (2021). Circular RNAs repertoire and expression profile during brassica rapa pollen development. Int. J. Mol. Sci. 22 (19), 10297. doi: 10.3390/ijms221910297 34638635 PMC8508787

[B6] Bardou F. Ariel F. Simpson C. G. Romero-Barrios N. Laporte P. Balzergue S.. (2014). Long noncoding RNA modulates alternative splicing regulators in arabidopsis. Dev. Cell 30 (2), 166–176. doi: 10.1016/j.devcel.2014.06.017 25073154

[B7] Borah P. Das A. Milner M. J. Ali A. Bentley A. R. Pandey R. (2018). Long non-coding RNAs as endogenous target mimics and exploration of their role in low nutrient stress tolerance in plants. Genes 9 (9), 459. doi: 10.3390/genes9090459 30223541 PMC6162444

[B8] Budak H. Kaya S. B. Cagirici H. B. (2020). Long non-coding RNA in plants in the era of reference sequences. Front. Plant Sci. 11, 276. doi: 10.3389/fpls.2020.00276 32226437 PMC7080850

[B9] Chen H. Wang T. Gong Z. Lu H. Chen Y. Deng F.. (2022). Low light conditions alter genome-wide profiles of circular RNAs in rice grains during grain filling. Plants 11 (9), 1272. doi: 10.3390/plants11091272 35567273 PMC9102277

[B10] Choi S.-W. Kim H.-W. Nam J.-W. (2019). The small peptide world in long noncoding RNAs. Brief Bioinform. 20 (5), 1853–1864. doi: 10.1093/bib/bby055 30010717 PMC6917221

[B11] Chu Q. Bai P. Zhu X. Zhang X. Mao L. Zhu Q.-H.. (2020). Characteristics of plant circular RNAs. Brief Bioinform. 21 (1), 135–143. doi: 10.1093/bib/bby111 30445438

[B12] Conn V. M. Hugouvieux V. Nayak A. Conos S. A. Capovilla G. Cildir G.. (2017). A circRNA from SEPALLATA3 regulates splicing of its cognate mRNA through r-loop formation. Nat. Plants 3 (5), 1–5. doi: 10.1038/nplants.2017.53 28418376

[B13] Csorba T. Questa J. I. Sun Q. Dean C. (2014). Antisense COOLAIR mediates the coordinated switching of chromatin states at FLC during vernalization. PNAS 111 (45), 16160–16165. doi: 10.1073/pnas.1419030111 25349421 PMC4234544

[B14] Ding J. Lu Q. Ouyang Y. Mao H. Zhang P. Yao J.. (2012). A long noncoding RNA regulates photoperiod-sensitive male sterility, an essential component of hybrid rice. PNAS 109 (7), 2654–2659. doi: 10.1073/pnas.1121374109 22308482 PMC3289353

[B15] Fang J. Zhang F. Wang H. Wang W. Zhao F. Li Z.. (2019). Ef-cd locus shortens rice maturity duration without yield penalty. PNAS 116 (37), 18717–18722. doi: 10.1073/pnas.1815030116 31451662 PMC6744900

[B16] Fan Y. Yang J. Mathioni S. M. Yu J. Shen J. Yang X.. (2016). PMS1T, producing phased small-interfering RNAs, regulates photoperiod-sensitive male sterility in rice. PNAS 113 (52), 15144–15149. doi: 10.1073/pnas.1619159114 27965387 PMC5206514

[B17] García-Gómez M. L. Castillo-Jiménez A. Martínez-García J. C. Álvarez-Buylla E. R. (2020). Multi-level gene regulatory network models to understand complex mechanisms underlying plant development. Curr. Opin. Plant Biol. 57, 171–179. doi: 10.1016/j.pbi.2020.09.004 33171396

[B18] Golicz A. A. Allu A. D. Li W. Lohani N. Singh M. B. Bhalla P. L. (2021). A dynamic intron retention program regulates the expression of several hundred genes during pollen meiosis. Plant Reprod. 34, 1–18. doi: 10.1007/s00497-021-00411-6 34019149

[B19] Golicz A. A. Bhalla P. L. Singh M. B. (2018). lncRNAs in plant and animal sexual reproduction. Trends Plant Sci. 23 (3), 195–205. doi: 10.1016/j.tplants.2017.12.009 29395831

[B20] Guria A. Sharma P. Natesan S. Pandi G. (2020). Circular RNAs–the road less traveled. Front. Mol. Biosci. 6, 146. doi: 10.3389/fmolb.2019.00146 31998746 PMC6965350

[B21] Haerizadeh F. Singh M. B. Bhalla P. L. (2006). Transcriptional repression distinguishes somatic from germ cell lineages in a plant. Science 313 (5786), 496–499. doi: 10.1126/science.1125526 16873660

[B22] Hansen T. B. Jensen T. I. Clausen B. H. Bramsen J. B. Finsen B. Damgaard C. K.. (2013). Natural RNA circles function as efficient microRNA sponges. Nature 495 (7441), 384–388. doi: 10.1038/nature11993 23446346

[B23] Heo J. B. Sung S. J. S. (2011). Vernalization-mediated epigenetic silencing by a long intronic noncoding RNA. Science 331 (6013), 76–79. doi: 10.1126/science.1197349 21127216

[B24] Huang L. Dong H. Zhou D. Li M. Liu Y. Zhang F.. (2018). Systematic identification of long non-coding RNA s during pollen development and fertilization in brassica rapa. Plant J. 96 (1), 203–222. doi: 10.1111/tpj.14016 29975432

[B25] Huang A. Zheng H. Wu Z. Chen M. Huang Y. (2020). Circular RNA-protein interactions: functions, mechanisms, and identification. Theranostics 10 (8), 3503. doi: 10.7150/thno.42174 32206104 PMC7069073

[B26] Itoh J.-I. Nonomura K.-I. Ikeda K. Yamaki S. Inukai Y. Yamagishi H.. (2005). Rice plant development: from zygote to spikelet. Plant Cell Physiol. 46 (1), 23–47. doi: 10.1093/pcp/pci501 15659435

[B27] Jarroux J. Morillon A. Pinskaya M. (2017). History, discovery, and classification of lncRNAs. Long Non Coding RNA Biol. (Singapore: Springer) 1008, 1–46. doi: 10.1007/978-981-10-5203-3_1 28815535

[B28] Kaikkonen M. U. Adelman K. (2018). Emerging roles of non-coding RNA transcription. Trends Biochem. Sci. 43 (9), 654–667. doi: 10.1016/j.tibs.2018.06.002 30145998

[B29] Kaufmann K. Chen D. (2017). “From genes to networks: characterizing gene-regulatory interactions in plants,” in Plant gene regulatory networks (New York, NY: Springer) 1629, 1–11. doi: 10.1007/978-1-4939-7125-1_1 28623575

[B30] Kiegle E. A. Garden A. Lacchini E. Kater M. M. (2018). A genomic view of alternative splicing of long non-coding RNAs during rice seed development reveals extensive splicing and lncRNA gene families. Front. Plant Sci. 9, 115. doi: 10.3389/fpls.2018.00115 29467783 PMC5808331

[B31] Kim D.-H. Sung S. (2017). Vernalization-triggered intragenic chromatin loop formation by long noncoding RNAs. Dev. Cell 40 (3), 302–312. e304. doi: 10.1016/j.devcel.2016.12.021 28132848 PMC5303624

[B32] Komiya R. Ohyanagi H. Niihama M. Watanabe T. Nakano M. Kurata N.. (2014). Rice germline-specific a rgonaute MEL 1 protein binds to phasi RNA s generated from more than 700 linc RNAs. Plant J. 78 (3), 385–397. doi: 10.1111/tpj.12483 24635777

[B33] Kong S. Tao M. Shen X. Ju S. (2020). Translatable circRNAs and lncRNAs: Driving mechanisms and functions of their translation products. Cancer Lett. 483, 59–65. doi: 10.1016/j.canlet.2020.04.006 32360179

[B34] Lamin-Samu A. T. Zhuo S. Ali M. Lu G. (2022). Long non-coding RNA transcriptome landscape of anthers at different developmental stages in response to drought stress in tomato. Genomics 114 (4), 110383. doi: 10.1016/j.ygeno.2022.110383 35550422

[B35] Li Z. Huang C. Bao C. Chen L. Lin M. Wang X.. (2015). Exon-intron circular RNAs regulate transcription in the nucleus. Nat. Struct. Mol. Biol. 22 (3), 256–264. doi: 10.1038/nsmb.2959 25664725

[B36] Li Y. Qin T. Dong N. Wei C. Zhang Y. Sun R.. (2019). Integrative analysis of the lncRNA and mRNA transcriptome revealed genes and pathways potentially involved in the anther abortion of cotton (Gossypium hirsutum l.). Genes 10 (12), 947. doi: 10.3390/genes10120947 31756984 PMC6947465

[B37] Li X. Shahid M. Q. Wen M. Chen S. Yu H. Jiao Y.. (2020). Global identification and analysis revealed differentially expressed lncRNAs associated with meiosis and low fertility in autotetraploid rice. BMC Plant Biol. 20 (1), 1–19. doi: 10.1186/s12870-020-2290-0 32075588 PMC7032005

[B38] Liu C.-X. Chen L.-L. (2022). Circular RNAs: Characterization, cellular roles, and applications. Cell 185, 2016–2034. doi: 10.1016/j.cell.2022.04.021 35584701

[B39] Liu Y. Su H. Zhang J. Liu Y. Feng C. Han F. (2020). Back-spliced RNA from retrotransposon binds to centromere and regulates centromeric chromatin loops in maize. PloS Biol. 18 (1), e3000582. doi: 10.1371/journal.pbio.3000582 31995554 PMC7010299

[B40] Liu H. Wang R. Mao B. Zhao B. Wang J. (2019). Identification of lncRNAs involved in rice ovule development and female gametophyte abortion by genome-wide screening and functional analysis. BMC Genomics 20 (1), 1–16. doi: 10.1186/s12864-019-5442-6 30691391 PMC6348626

[B41] Lucero L. Ferrero L. Fonouni-Farde C. Ariel F. (2021). Functional classification of plant long noncoding RNAs: a transcript is known by the company it keeps. New Phytol. 229 (3), 1251–1260. doi: 10.1111/nph.16903 32880949

[B42] Luo X. Yin M. He Y. (2021). Molecular genetic understanding of photoperiodic regulation of flowering time in Arabidopsis and soybean. Int. J. Mol. Sci. 23 (1), 466. doi: 10.3390/ijms23010466 35008892 PMC8745532

[B43] Memczak S. Jens M. Elefsinioti A. Torti F. Krueger J. Rybak A.. (2013). Circular RNAs are a large class of animal RNAs with regulatory potency. Nature 495 (7441), 333–338. doi: 10.1038/nature11928 23446348

[B44] Meng X. Li A. Yu B. Li S. (2021). Interplay between miRNAs and lncRNAs: Mode of action and biological roles in plant development and stress adaptation. Comput. Struct. Biotechnol. J. 19, 2567–2574. doi: 10.1016/j.csbj.2021.04.062 34025943 PMC8114054

[B45] Nonomura K.-I. Morohoshi A. Nakano M. Eiguchi M. Miyao A. Hirochika H.. (2007). A germ cell–specific gene of the ARGONAUTE family is essential for the progression of premeiotic mitosis and meiosis during sporogenesis in rice. Plant Cell 19 (8), 2583–2594. doi: 10.1105/tpc.107.053199 17675402 PMC2002623

[B46] Okada T. Singh M. B. Bhalla P. L. (2005). Expressed sequence tag analysis of *Lilium longiflorum* generative cell. Plant Cell Physiol. 47 (6), 698–705. doi: 10.1093/pcp/pcj040 16571618

[B47] Okada T. Singh M. B. Bhalla P. L. (2007). Transcriptome profiling of *Lilium longiflorum* generative cells by cDNA microarray. Plant Cell Rep. 26 (7), 1045–1052. doi: 10.1007/s00299-006-0300-9 17245599

[B48] Panni S. Lovering R. C. Porras P. Orchard S. (2020). Non-coding RNA regulatory networks. Biochim. Biophys. Acta (BBA)-Gene Regul. Mech. 1863(6), 194417. doi: 10.1016/j.bbagrm.2019.194417 31493559

[B49] Philips A. Nowis K. Stelmaszczuk M. Jackowiak P. Podkowiński J. Handschuh L.. (2020). Expression landscape of circRNAs in *arabidopsis thaliana* seedlings and adult tissues. Front. Plant Sci. 11, 576581. doi: 10.3389/fpls.2020.576581 33014000 PMC7511659

[B50] Rai M. I. Alam M. Lightfoot D. A. Gurha P. Afzal A. J. (2019). Classification and experimental identification of plant long non-coding RNAs. Genomics 111 (5), 997–1005. doi: 10.1016/j.ygeno.2018.04.014 29679643

[B51] Ransohoff J. D. Wei Y. Khavari P. A. (2018). The functions and unique features of long intergenic non-coding RNA. Nat. Rev. Mol. 19 (3), 143–157. doi: 10.1038/nrm.2017.104 PMC5889127 29138516

[B52] Russell S. D. Gou X. Wong C. E. Wang X. Wei X. Bhalla P. L.. (2012). Genomic profiling of rice sperm cell transcripts reveals conserved and distinct elements in the flowering plant male germ lineage. New Phytol. 195 (3), 560–573. doi: 10.1111/j.1469-8137.2012.04199.x 22716952

[B53] Ryu C.H. Lee S. Cho L.H. Kim S.L. Lee Y.S. Choi S.C.. (2009). OsMADS50 and OsMADS56 function antagonistically in regulating long day (LD)‐dependent flowering in rice. Plant, Cell Environ. 32 (10), 1412–1427. doi: 10.1111/j.1365-3040.2009.02008.x 19558411

[B54] Sebastian-delaCruz M. Gonzalez-Moro I. Olazagoitia-Garmendia A. Castellanos-Rubio A. Santin I. (2021). The role of lncRNAs in gene expression regulation through mRNA stabilization. Non-coding RNA 7 (1), 3. doi: 10.3390/ncrna7010003 33466464 PMC7839045

[B55] Seo J. S. Sun H.-X. Park B. S. Huang C.-H. Yeh S.-D. Jung C.. (2017). ELF18-INDUCED LONG-NONCODING RNA associates with mediator to enhance expression of innate immune response genes in arabidopsis. Plant Cell 29 (5), 1024–1038. doi: 10.1105/tpc.16.00886 28400491 PMC5466027

[B56] Sharma N. Russell S. C. Bhalla P. L. Singh M. B. (2011). Putative cis-regulatory elements in genes highly expressed in rice sperm cells. BMC Res. Notes 4, 319. doi: 10.1186/1756-0500-4-319 21892935 PMC3224587

[B57] Shin W. J. Nam A. H. Kim J. Y. Kwak J. S. Song J. T. Seo H. S. (2022). Intronic long noncoding RNA, RICE FLOWERING ASSOCIATED (RIFLA), regulates OsMADS56-mediated flowering in rice. Plant Sci. 320, 111278. doi: 10.1016/j.plantsci.2022.111278 35643617

[B58] Shi D.-Q. Yang W.-C. (2011). Ovule development in arabidopsis: progress and challenge. Curr. Opin. Plant Biol. 14 (1), 74–80. doi: 10.1016/j.pbi.2010.09.001 20884278

[B59] Singh M. B. Bhalla P. L. (2007). Control of male germ cell development in flowering plants. BioEssays 29 (11), 1124–1132. doi: 10.1002/bies.20660 17935220

[B60] Singh M. B. Bhalla P. L. Russell S. D. (2008). Molecular repertoire of flowering plant male germ cells. Sex Plant Reprod. 21 (1), 27–36. doi: 10.1007/s00497-008-0067-y

[B61] Teotia S. Tang G. (2015). To bloom or not to bloom: role of microRNAs in plant flowering. Mol. Plant 8 (3), 359–377. doi: 10.1016/j.molp.2014.12.018 25737467

[B62] Wang Y. Luo X. Sun F. Hu J. Zha X. Su W.. (2018). Overexpressing lncRNA LAIR increases grain yield and regulates neighbouring gene cluster expression in rice. Nat. Commun. 9 (1), 1–9. doi: 10.1038/s41467-018-05829-7 30158538 PMC6115402

[B63] Wang M. Wu H.-J. Fang J. Chu C. Wang X.-J. (2017). A long noncoding RNA involved in rice reproductive development by negatively regulating osa-miR160. Sci. Bull. 62 (7), 470–475. doi: 10.1016/j.scib.2017.03.013 36659255

[B64] Wang Y. Xiong Z. Li Q. Sun Y. Jin J. Chen H.. (2019). Circular RNA profiling of the rice photo-thermosensitive genic male sterile line wuxiang s reveals circRNA involved in the fertility transition. BMC Plant Biol. 19 (1), 1–16. doi: 10.1186/s12870-019-1944-2 31382873 PMC6683460

[B65] Wang Y. Zhang H. Li Q. Jin J. Chen H. Zou Y.. (2021). Genome-wide identification of lncRNAs involved in fertility transition in the photo-thermosensitive genic Male sterile rice line wuxiang s. Front. Plant Sci. 11, 580050. doi: 10.3389/fpls.2020.580050 33519839 PMC7840536

[B66] Waseem M. Liu Y. Xia R. (2020). Long non-coding RNAs, the dark matter: an emerging regulatory component in plants. Int. J. Mol. Sci. 22 (1), 86. doi: 10.3390/ijms22010086 33374835 PMC7795044

[B67] Wierzbicki A. Blevins T. Swiezewski S. (2021). Long noncoding RNAs in plants. Annu. Rev. Plant Biol. 72 (1), 245–271. doi: 10.1146/annurev-arplant-093020-035446 33752440

[B68] Wong C.E. Singh M.B. and Bhalla P.L. (2013). The dynamics of soybean leaf and shoot apical meristem transcriptome undergoing floral initiation process. PLoS One 8 (6), e65319. doi: 10.1371/journal.pone.0065319 23762343 PMC3675103

[B69] Wu S. Xu H. Zhang R. Wang X. Yang J. Li X.. (2022). Circular RNA circLAMA3 inhibits the proliferation of bladder cancer by directly binding an mRNA. Mol. Therapy-Oncolytics 24, 742–754. doi: 10.1016/j.omto.2022.02.020 PMC8908064 35317525

[B70] Xiao M.-S. Ai Y. Wilusz J. E. (2020). Biogenesis and functions of circular RNAs come into focus. Trends Cell Biol. 30 (3), 226–240. doi: 10.1016/j.tcb.2019.12.004 31973951 PMC7069689

[B71] Yamaguchi A. Abe M. (2012). Regulation of reproductive development by non-coding RNA in Arabidopsis: to flower or not to flower. 125 (6), 693–704. doi: 10.1007/s10265-012-0513-7 PMC3485539 22836383

[B72] Yu Y. Zhang Y. Chen X. Chen Y. (2019). Plant noncoding RNAs: hidden players in development and stress responses. Annu. Rev. Cell Dev. 35, 407. doi: 10.1146/annurev-cellbio-100818-125218 PMC8034839 31403819

[B73] Zhang J.-P. Yu Y. Feng Y.-Z. Zhou Y.-F. Zhang F. Yang Y.-W.. (2017). MiR408 regulates grain yield and photosynthesis via a phytocyanin protein. Plant Physiol. 175 (3), 1175–1185. doi: 10.1104/pp.17.01169 28904074 PMC5664482

[B74] Zhang Y.-C. Liao J.-Y. Li Z.-Y. Yu Y. Zhang J.-P. Li Q.-F.. (2014). Genome-wide screening and functional analysis identify a large number of long noncoding RNAs involved in the sexual reproduction of rice. Genome Biol. 15 (12), 1–16. doi: 10.1186/s13059-014-0512-1 PMC4253996 25517485

[B75] Zhang P. Li S. Chen M. (2020). Characterization and function of circular RNAs in plants. Front. Mol. Biosci. 7, 91. doi: 10.3389/fmolb.2020.00091 32509801 PMC7248317

[B76] Zhang J. Li C. Wu C. Xiong L. Chen G. Zhang Q.. (2006). RMD: a rice mutant database for functional analysis of the rice genome. Nucleic Acids Res. 34 (suppl_1), D745–D748. doi: 10.1093/nar/gkj016 16381972 PMC1347379

[B77] Zhao J. Ajadi A. A. Wang Y. Tong X. Wang H. Tang L.. (2020). Genome-wide identification of lncRNAs during rice seed development. Genes 11 (3), 243. doi: 10.3390/genes11030243 32110990 PMC7140839

[B78] Zhao W. Cheng Y. Zhang C. You Q. Shen X. Guo W.. (2017). Genome-wide identification and characterization of circular RNAs by high throughput sequencing in soybean. Sci. Rep. 7 (1), 1–11. doi: 10.1038/s41598-017-05922-9 28717203 PMC5514102

[B79] Zhao W. Chu S. Jiao Y. (2019). Present scenario of circular RNAs (circRNAs) in plants. Front. Plant Sci. 10, 379. doi: 10.3389/fpls.2019.00379 31001302 PMC6454147

[B80] Zhou J. Yuan M. Zhao Y. Quan Q. Yu D. Yang H.. (2021a). Efficient deletion of multiple circle RNA loci by CRISPR‐Cas9 reveals Os06circ02797 as a putative sponge for OsMIR408 in rice. Plant Biotechnol. J. 19 (6), 1240–1252. doi: 10.1111/pbi.13544 33440058 PMC8196656

[B81] Zhou Y.-F. Zhang Y.-C. Sun Y.-M. Yu Y. Lei M.-Q. Yang Y.-W.. (2021b). The parent-of-origin lncRNA MISSEN regulates rice endosperm development. Nat. Commun. 12 (1), 1–14. doi: 10.1038/s41467-021-26795-7 34764271 PMC8585977

